# Modeling a population of retinal ganglion cells with restricted Boltzmann machines

**DOI:** 10.1038/s41598-020-73691-z

**Published:** 2020-10-06

**Authors:** Riccardo Volpi, Matteo Zanotto, Alessandro Maccione, Stefano Di Marco, Luca Berdondini, Diego Sona, Vittorio Murino

**Affiliations:** 1 Naver Labs Europe, Meylan, France; 2 Istituto Italiano di Tecnologia, Genoa, Italy; 3 Università degli Studi dell’Aquila, L’Aquila, Italy; 4 Università di Verona, Verona, Italy

**Keywords:** Neural encoding, Machine learning

## Abstract

The retina is a complex circuit of the central nervous system whose aim is to encode visual stimuli prior the higher order processing performed in the visual cortex. Due to the importance of its role, modeling the retina to advance in interpreting its spiking activity output is a well studied problem. In particular, it has been shown that latent variable models can be used to model the joint distribution of Retinal Ganglion Cells (RGCs). In this work, we validate the applicability of Restricted Boltzmann Machines to model the spiking activity responses of a large a population of RGCs recorded with high-resolution electrode arrays. In particular, we show that latent variables can encode modes in the RGC activity distribution that are closely related to the visual stimuli. In contrast to previous work, we further validate our findings by comparing results associated with recordings from retinas under normal and altered encoding conditions obtained by pharmacological manipulation. In these conditions, we observe that the model reflects well-known physiological behaviors of the retina. Finally, we show that we can also discover temporal patterns, associated with distinct dynamics of the stimuli.

## Introduction

Retinal Ganglion Cells (RGCs) play a key role in the visual system, being the output of the retina circuit and the connection between the retina and the areas of the brain dedicated to the high-level processing of the visual stimuli. For this reason, understanding how the visual information is encoded in the RGC population spiking activity is a very interesting problem in neuroscience, with relevant implications for retinal prostheses. Recent work^1^ has shown that latent factor models can encode regularities in the population spiking activity that are associated with the stimuli shown to the retina, in an unsupervised fashion. It has been further shown that a specific latent factor model, namely Restricted Boltzmann Machines^2^ (RBMs), can learn latent factors in a joint distribution that preserve the similarity between visual stimuli shown to the retina^3^. RBMs are latent variable models capable of learning the joint distribution of a set of continuous observed random variables and a set of binary unobserved random variables (often referred to as *latent units*). Once the model is trained, it allows to represent regularities in the observed inputs through the inferred values of the latent binary variables. A significant advantage of using RBMs is that the connections between visible units do not have to be explicitly modeled. Indeed, the hidden units independently discover the relationships between them.

In this work, we further validate the applicability of RBMs to model the joint distribution of RGC spiking activity evoked by visual stimuli. While previous work^3^ has shown that RBMs allow to learn a good “neural metric”, our main contribution is showing that this class of models is able to discover patterns associated with the stimuli shown to the retina, in a fully unsupervised fashion, through a series of experiments. With “unsupervised”, we intend here without giving any information about the stimuli to the model. We show that, by modeling the population distribution with RBMs, the model’s specific hidden states are activated by firing rate samples associated with specific stimuli (for example, gratings in a specific position), and different hidden units are activated by more complex activity, that resembles that of receptive fields.

The core idea behind this approach is that the joint population presents a series of modes in their behaviour, and that these modes are strongly correlated with the proposed stimuli. By “modes”, we are referring to the regularities in the population activity. We are making the assumption that the visual information is encoded in the spiking rate of the RGCs, so when we refer to “population activity” we are always referring to the firing rate of a large number of RGCs.

**Figure 1:**
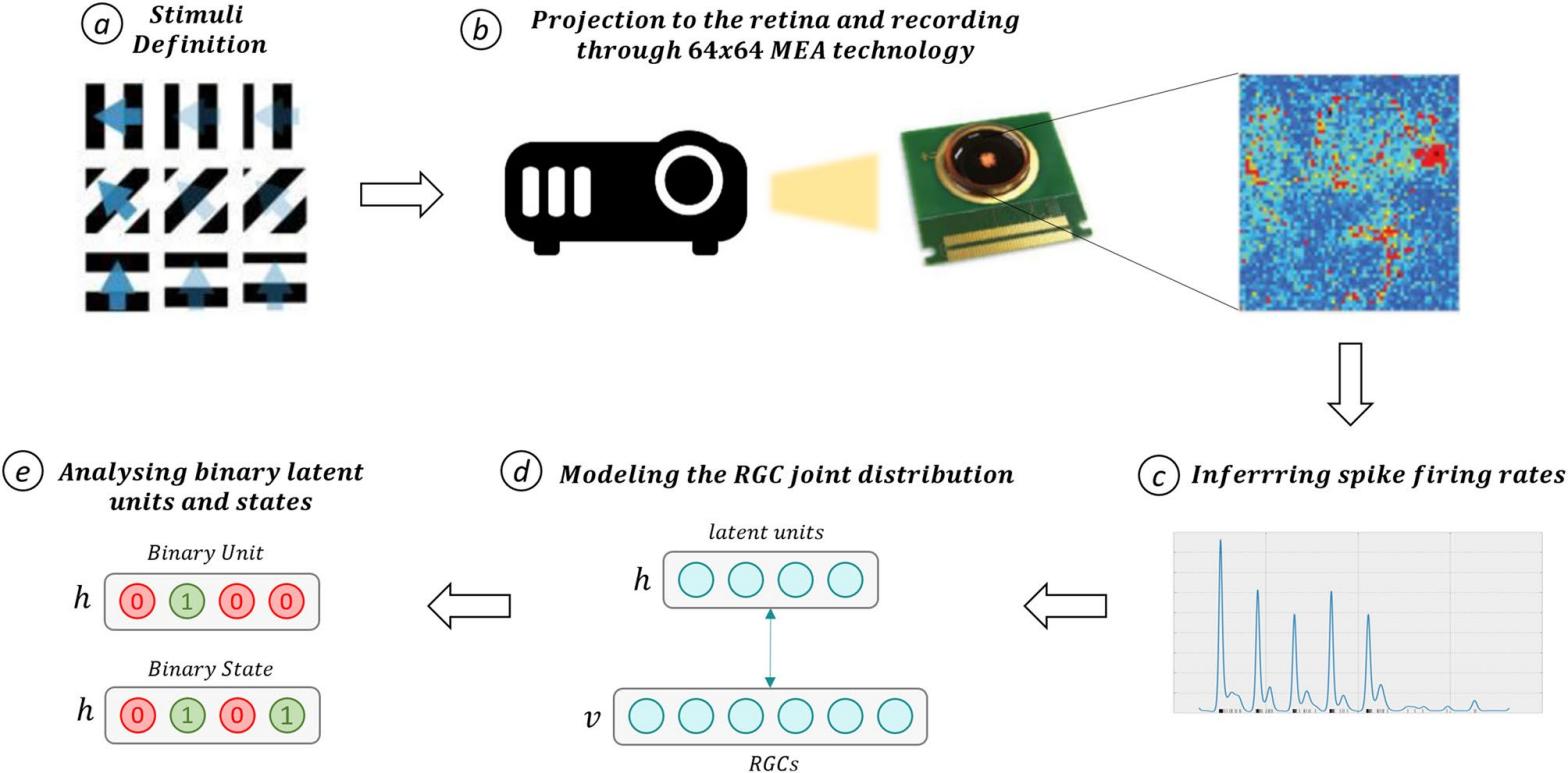
Setup that characterizes our experiments. (**a**) The stimuli to be shown are defined: they can be simple (gratings) or more complex (natural images). (**b**) Such stimuli are shown to a mouse retina through a projector; the RGC signals are recorded through a High Density $64 \times 64$ MEA chip. (**c**) Before being able to process the data, the firing rates need to be inferred from the spike trains; this step is carried out through log-Gaussian Cox processes. (**d**) Data is then processed by our models, which are trained as described in text. The visible units which define layer *v* take in input firing rate samples, and map those in a latent representation (layers *h*). (**e**) With a trained model, we can extract the latent representation related to each firing rate sample and analyze what different binary units/states have encoded, through the visualization technique described in text. In summary, we retrieve the stimuli associated with each firing rate sample that activate a particular unit/state and average them.

Figure 1 shows a schematic view of the pipeline we follow to carry out our analysis. New generations of Multi-Electrode Array (MEA) based on CMOS-technology^4^ are unique tools to study neural populations, as these devices allow to simultaneously record the activity of hundreds of RGCs activated by visual stimuli. We acquired different datasets, obtained through CMOS-MEAs with $64\times 64$ channels, coupled with a projector presenting visual stimuli to explanted mouse retinas mounted and maintained on the chip. After recordings, we modeled the joint distribution of the firing rates with mean-covariance Restricted Boltzmann Machines^5,6^ (mcRBMs) , and—to detect *temporal* patterns—with conditional Restricted Boltzmann Machines^7^ (cRBMs)

We validated our hypothesis for both simple stimuli (gratings) and more complex ones (natural scenes). As a starting point, we investigate if the modes—namely, the regularities—in the firing rate distribution associated with the visual stimuli can be discovered through the latent units of RBMs. To this aim, we designed an experiment where square gratings moving in eight directions were shown to an adult mouse retina. Further, we investigated how these models respond to different levels of retina impairment induced by pharmacological manipulation of the GABA receptors of the entire retina. In particular, we investigated whether the retrieved modes reflect inner coherence in the processing of the retina, and figure out whether the decreased spatio-temporal precision of the encoding expected from physiology^8,9^ is captured by the model. To shed light on this question, we report results associated with a study where the RGC activity of an adult mouse retina was recorded when stimulated with different types of moving square gratings, both in normal conditions and after blocking *GABA* receptors ($GABA_C$ at first, and then also $GABA_A$ and $GABA_B$). As expected, the ability of the model to learn regularities in the data decreases after having increased the blockade of GABA receptors. This is because pharmacologically induced impairment leads to an unreliable population code. As a further step, we were interested in evaluating the efficiency of our models when applied to population activities recorded from a retina stimulated with more complex visual stimuli. To do so, we have chosen to use a series of natural images representing a movement of the visual field over a brick wall. Even with such more complex stimuli, the models detected stimuli-associated modes. Finally, the possibility of retrieving temporal patterns related to different dynamics of the stimuli is investigated, by modeling the RGC joint distribution. We address this task by modeling the RGC population activity with a specific type of RBM (the *conditional* RBM^7^), which allows to model also the history of the input.

In summary, in this work we address:

1. Whether RBMs are able to find modes in the RGC firing rate associated with simple and complex visual stimuli shown to the retina;
2. How the impairment of the retina is reflected in the performance of the model;
3. Whether temporal modes associated with the dynamics of the stimuli are detectable.

In the rest of this section, related works are detailed.

### Related work

Different works address the problem of decoding RGC activity. Bialek et al.^10^ introduce the problem of decoding the activity of a movement-sensitive neuron in the visual system of flyes. Warland et al.^11^ show that stimuli (in their case, photopic, spatially uniform, temporally broadband flicker) can be decoded from spike trains, and that the decoding performance highly improves considering multiple spike trains. Pillow et al.^12^ evaluate a decoding approach based on correlations among different neurons’ activity. Marre et al.^13^ define a linear decoder trained over a population of RGCs stimulated with a moving bar, and they show that the position of the bar can be decoded from the neural activity with high accuracy. Botella-Soler et al.^14^ address the problem of reconstructing the stimuli proposed from RGC spike activities, reporting promising results. In summary, related works^10–14^ address the problem of understanding the relationship between RGC population activity and stimuli as a *supervised* task, namely, explicitly defining the connection between the stimuli shown to the retina and the corresponding RGC activity during the model training procedure.

Differently, we are interested in models able to discover patterns in an *unsupervised* fashion, in order to find regularities in the retina states in a fully data-driven manner. The discovered regularities can be related to the stimuli with a posterior analysis.

The application of unsupervised learning models to retinal activity is not new to this work. Trying to infer the latent structure of RGCs, Linderman et al.^15^ show that cell types and receptive field center locations can be unsupervisely learned from spike trains, using a Generalized Linear Model^16^. Tkacik et al.^17^ show that maximum-entropy models can be used to model the correlations among different neurons. Prentice et al.^1^ model the retinal population with models able to encode different modes, and show that these modes are closely correlated with the stimuli proposed to the retina. More recently, Gardella et al.^3^ have shown that RBMs can be used to learn a neural metric that preserves the similarity of stimuli shown to the retina.

In this work, we further validate the ability of RBMs to model the RGC population activity in a plethora of different scenarios, and by exploiting high-resolution MEAs for large-scale rerecording of light-evoked RGCs activity. In particular, while Gardella et al.^3^ evaluate the possibility of using RBM hidden states as a neural metric, we investigate the ability of RBMs to retrieve common patterns in the electrical activity and analyze their relationship with the stimuli showed to the retina, demonstrating that this model can retrieve activity patterns related to similar stimuli. Furthermore, we extend this analysis to the dynamic case, showing that conditional RBMs can retrieve temporal patterns associated with similar stimuli dynamics.

Finally, a novel aspect of this work is challenging the model to represent different levels of impairments of visual information encoding as obtained by pharmacological manipulation of GABA receptors. Our results show the relationship between the behavior of the model and different levels of impairment of the retina (artificially induced by blocking GABA receptors).

## Materials and methods

### Ethics statement

All experiments and procedures for preparing retinas from mice used in this work were approved by the IIT Animal Use Committee and by the Italian Ministry of Health (Authorization No. 110/2014-PR of the 19th of December 2014), and follow the Guide for the Care and Use of Laboratory Animals of the European Community Council Directives.

### Experimental setup

All experiments were carried out with High Density MEA technology with $64 \times 64$ channels, arranged in a square lattice and separated by 42 m. Sampling rates were in the range of 7–8 kHz and the stimuli were shown to the retina with a frequency of 30 Hz. The detailed description of the system is reported in Berdondini et al.^4^ and Maccione et al.^18^.

After every experiment, to separate the activities of the different cells, we performed spike sorting using Plexon Offline Sorter (http://www.plexon.com/products/offline-sorter) and then manually refined the results. The firing rate was inferred from the recorded spike trains via log-Gaussian Cox processes^19^: being doubly stochastic, we believe it represents a good model to deal with the stochasticity of RGC activity. In order to model only neurons that carry information relative to the stimuli, we only used the neurons presenting a time-varying firing rate. To this end, we removed all the neurons for which the difference between the maximum and the minimum values of the derivative of the firing rate was below a fixed threshold. In this way, we removed both silent neurons and neurons with a tonic firing rate. We dropped those neurons under the assumption that they do not carry any information to characterize the stimuli.

### Restricted Boltzmann machines

Restricted Boltzmann Machines^20^ (RBMs) are energy-based, latent variable models capable of learning in an unsupervised fashion the joint distribution of a set of observed random variables and a set of binary unobserved random variables (often referred to as *latent* variables). In this model, the input variables are connected with stochastic, binary feature detectors, through symmetric weights.

The energy function associated with the joint configuration of visible and hidden variables, in case of binary input and hidden units, is defined by

1 $$\begin{aligned} E(\mathbf{v} ,\mathbf{h} ) = - \mathbf{a} ^T\mathbf{v} - \mathbf{b} ^T\mathbf{h} -\mathbf{v} ^T\mathbf{W} {} \mathbf{h} , \end{aligned}$$

where **v**,**h** are the values associated with visible and hidden units, respectively; **a**,**b** are the biases associated with those units, and **W** is a weight matrix. The energy is related to the probability density of visible and hidden units via

2 $$\begin{aligned} P(\mathbf{v} ,\mathbf{h} ) \propto e^{-E(\mathbf{v} ,\mathbf{h} )}. \end{aligned}$$

In case we want to model real-valued inputs, visible units are replaced with linear units with independent Gaussian noise, and the energy function becomes

3 $$\begin{aligned} E(\mathbf{v} ,\mathbf{h} ) = - \frac{1}{2}(\mathbf{v} -\mathbf{a} )^T(\mathbf{v} - \mathbf{a} ) - \mathbf{b} ^T\mathbf{h} -\mathbf{v} ^T\mathbf{W} {} \mathbf{h} , \end{aligned}$$

assuming unitary standard deviation of the Gaussian noise associated with visible units.

In this work, each visible unit is associated with a different ganglion cell, and the values fed in input are the firing rate values of the RGCs at different time steps. We can train such energy-based models using an algorithm called contrastive divergence^21,22^. Once the model is trained, the bi-partition allows to represent regularities in the observed inputs through the inferred values of the latent binary variables. Note that we have reported only the details necessary to a basic understanding of RBMs, the technical report by Hinton^2^ gives more a detailed explanation

We report in the following the two particular types of RBMs used in our experimentation, namely the *mean-covariance* RBM^5,6^ (mcRBM) and the *conditional* RBM^7^ (cRBM).

### Mean-covariance restricted Boltzmann machines

The mcRBM model^5,6^ is a modified version of the standard RBM, where the hidden units are divided in two different sets: mean units and precision units. The former is used to model the mean value of the inputs, the latter to explicitly modeling the covariance between the observed variables. The presence of this second set allows to obtain a much better fit of the data distribution than what can be achieved with simpler models like Gaussian RBMs. The mathematical detailes are reported in the Supplementary Material. An important property of mcRBMs is that, conditioning on the latent variables, the observed ones are approximately jointly Gaussian distributed^6^. The mean and covariance of these Gaussians are defined by the specific values of the hidden units. This means that each of the possible binary vectors representing the latent variable values is associated with one mode of the joint distribution of the inputs. This aspect makes mcRBM a very good model for our purposes of finding the regularities associated with visual stimuli shown to the retina.

### Conditional restricted Boltzmann machines

RBMs and mcRBMs are not able to model the dynamics of the input, since one frame is processed at the time. The cRBM^7^ variant allows to model the temporal evolution of the input, by simultaneously considering different visible layers, each one associated with delayed versions of the input. The cRBM model includes two additional types of connections: autoregressive connections that link delayed input to the present one and connections between the hidden units and the history of the input. The new connections can be interpreted as a dynamic bias, and in this way the learning algorithm defined for RBMs^21^ does not change, it simply needs to be extended to these new weights, too. See Taylor et al.^7^ for a more technical explanation of the model.

### Visualizing the modes

To understand which visual features the RBM states (and units) have encoded, we propose a technique closely related to spike-triggered average^23^ (STA), implemented as follows: (i) one binary state/unit is selected; (ii) the firing rate samples that activate that particular state/unit are retrieved; (iii) the stimuli associated with those samples are identified and averaged. Following this procedure, we can understand whether the regularities encoded by the RBM states are associated with specific stimuli or groups of stimuli.

This technique can be applied in the same way also to cRBMs. In this case, we need to average the stimuli sequences, preserving the temporal order. We obtain sequences of averages, that provide information on the time-changing input frames that are associated with specific modes in the firing rate distribution. In this way, we can visually assess the dynamics of the stimuli related to different configurations of the hidden layer.

### Model evaluation

While the visualization technique described above gives a qualitative view of what the models have learned, we lack of a quantitative measure to evaluate the meaningfulness of the modes learned by the models. An objective measure is particularly useful when comparing the model performance when trained on data associated with different levels of impairment of the retina, to evaluate which model better fits the data.

We rely on information theory to evaluate whether the modes encoded by RBMs are coherently associated with particular visual stimuli. Specifically, we compute the mutual information between the stimuli proposed to the retina and the binary states learned by the RBMs. This metric provides a quantitative measure which tells us how well the model has encoded the regularities associated with the visual stimuli. The mutual information between two discrete random variables is defined by the following equation

4 $$\begin{aligned} I(X;Y) = \sum _{y \in Y}\sum _{x \in X}p(x,y)log\Big (\frac{p(x,y)}{p(x)p(y)}\Big ) \end{aligned}$$

where *X* and *Y* are the whole sets of stimuli and binary states, respectively. Since *I* is maximum when each state is associated with a different stimulus, we divide this term by the entropy of the stimuli, obtaining the normalized mutual information, i.e., a measure in the range 0–1.

## Results

**Figure 2:**
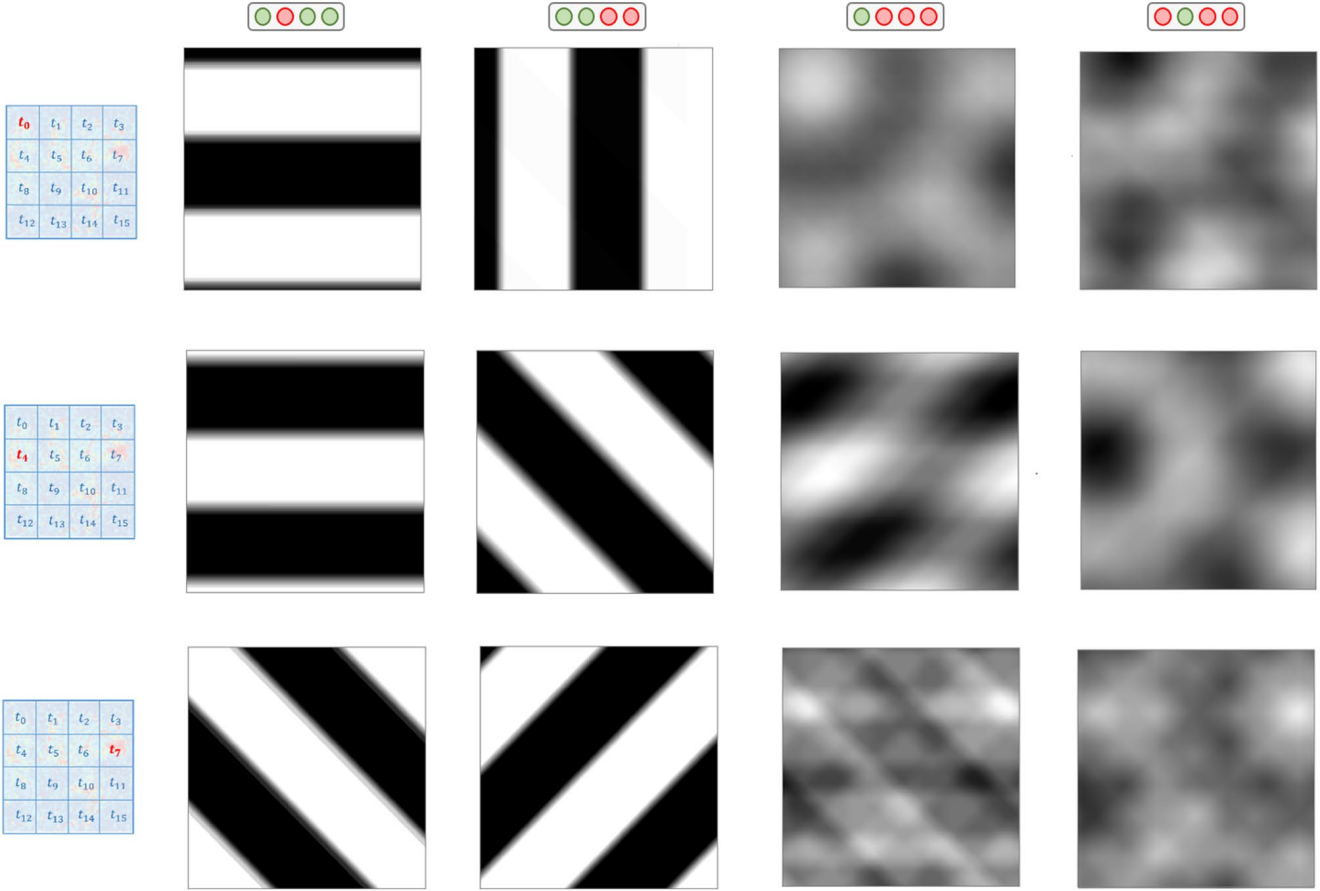
Results related to Experiment 1. Each row is associated with a different patch of the MEA chip, from which neurons were recorded during the experiments. Top-down: patches t0, t4, t7. The first two columns represent averages over stimuli associated with different binary states, the last two columns represent averages over stimuli associated with different binary units.

In *Experiment 1*, the population activity related to grating stimulation is modeled with mcRBMs^5,6^, to assess whether this class of models can retrieve modes associated with visual features. In *Experiment 2*, model performances associated with different levels of impairment are compared, to investigate if the model performances reflects the inner coherence of the retina. *Experiment 3* presents the same analysis of the first one, but with more complex stimuli. In *Experiments 4*, the population activity is modeled with cRBMs^7^, to understand whether also temporal modes can be retrieved. In order to manage the complexity of the task, the MEA chip was divided in $4\times 4$ non-overlapping regions (each patch composed by $16\times 16$ electrodes), further referred to as $t_{i}$, $i = 0-15$ (as shown in Fig. 2, left). The results are discussed in the next section.

### Experiment 1: heterogeneous grating stimulation

We stimulated a P38 stage mouse retina with gratings with different orientations. Specifically, a moving square grating with 800 m-wide bars was presented to the retina in 8 different orientations: $0^{\circ }$, $45^{\circ }$, $90^{\circ }$, $135^{\circ }$, $180^{\circ }$, $225^{\circ }$, $270^{\circ }$ and $315^{\circ }$. For each orientation, ten repetitions of the stimuli were carried out. Figure 1a shows different samples of stimuli. Totally, 3923 neurons were recorded, but only 1344 were used for the experiments, following the selection described above.

Figure 2 shows some examples of the visual representation associated with different hidden states or units, obtained through the STA-based representation described in previous section. Each row reports results related to neurons recorded from different patches of the chip. From top to bottom: t0 (114 neurons), t4 (132 neurons) and t7 (61 neurons)—see the locations of the patches in the squares reported on the left of Fig. 2. The first two columns contain images associated with different binary states, the last two columns contain images associated with different binary units.

### Experiment 2: effect of GABA blockage

**Figure 3:**
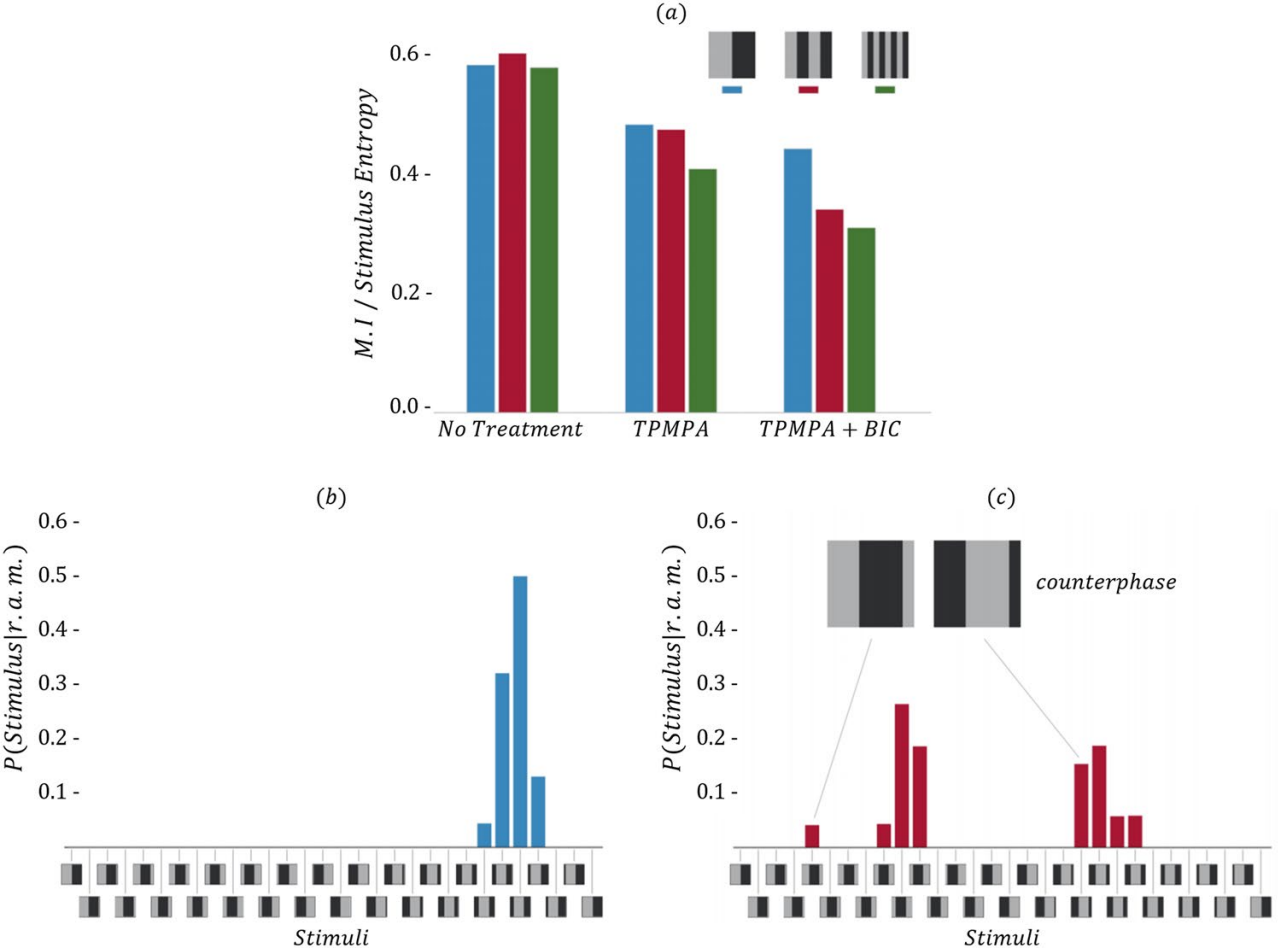
Results related to Experiment 2. Panel (**a**) shows the mutual information associated with (left to right) retina without GABA blockage, retina with $GABA_C$ blockage and retina with also $GABA_A$ and $GABA_B$ blockage. Different colors are associated with gratings with different spatial frequency: 0.011, 0.023 and 0.045 cycles/degree in blue, red and green, respectively. Panels (**b**,**c**) show the distribution of the stimuli associated with a single latent state. While in retina without impairment a good match is found (**b**), blocking GABA receptors results in a mismatch (**c**): stimuli in counterphase are encoded in the same state.

We recorded the activity of an adult mouse retina (P45 stage) when stimulated with three different moving square gratings in three different recording conditions. At first, the retina in normal conditions was subject to three stimuli with different frequencies:

1. Square grating at 0.011 cycles/degree.
2. Square grating at 0.023 cycles/degree.
3. Square grating at 0.045 cycles/degree.

All the stimulation protocols had temporal frequency of 1 Hz, Michelson contrast 0.5 and mean luminance $1.36 cd/m^2$. The following stages of the experiment consisted in repeating the same stimulations under two different pharmacological treatments aimed at progressively blocking *GABA* receptors and hence impairing the functional behaviour of the inhibitory circuitry. For this, we rely on TPMPA and Bicuculline, which are GABA receptors antagonists, selective for $GABA_C$ and $GABA_A$/$GABA_B$ receptors, respectively. Specifically, TPMPA 150*M* was added first to block $GABA_C$ receptors and Bicuculline 10*M* was successively added to also block $GABA_A$ and $GABA_B$ receptors.

To quantitatively evaluate the impairment, we calculated the normalized Mutual Information (MI) between the states learned by the model and the stimuli proposed. As expected, GABA blockage resulted in a lower MI between the states and the stimuli. Results are reported in Fig. 3a, which shows the MI scores associated with the different levels of impairment (no treatment, TPMPA and TPMPA $+$ Bicuculline), with three different types of grating stimuli in blue, red and green. Figure 3b,c show the distributions of the stimuli associated with arbitrarily chosen states for the retina in normal conditions and after having impaired the circuitry, respectively.

We report one single example in this work, but similar behaviors were detected throughout the collection of states learned by the mcRBM.

### Experiment 3: natural scenes

**Figure 4:**
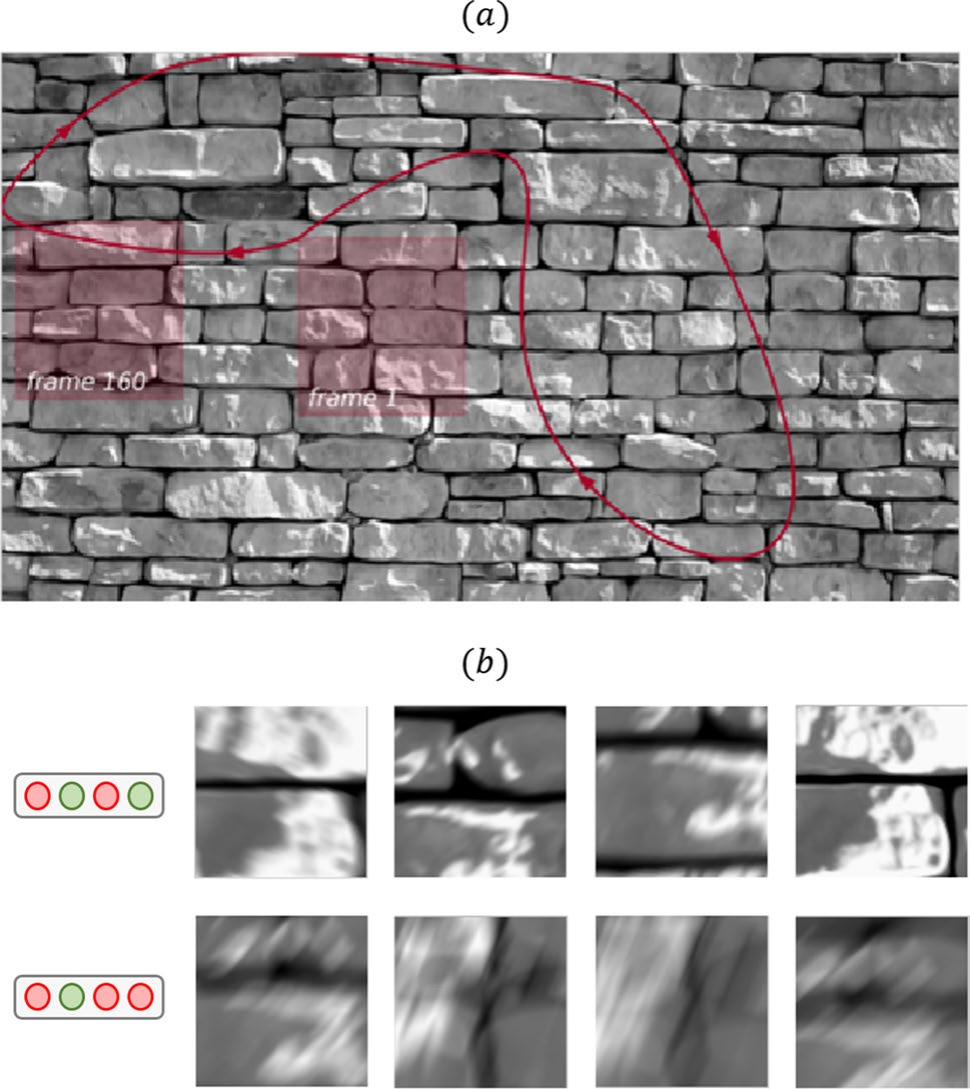
Results related to Experiment 3. Panel (**a**) shows the trajectory followed to show natural scene stimuli to the retina during Experiment 3. Panel (**b**) shows visual features encoded by different binary states (top) and binary units (bottom). While visual features extracted via hidden states and units appear more similar than what we could observe, e.g., in Fig. 2, it can still be observed that units reflect more generic visual features (they are more blurred, because they aggregate more stimuli).

Figure 4a reports the stimuli shown to a P38 stage mouse retina, obtained by defining a trajectory of a sliding window over an image of a brick wall. The firing rate population activity inferred from spike trains was modeled through mcRBMs following the same procedures detailed for *Experiment 1*.

Figure 4b shows some visual features obtained via our STA-like visualization technique. The first row shows an example of STA associated with single binary states, the second row, instead, shows an example of average stimuli associated with different binary units.

### Experiment 4: modeling population dynamics

**Figure 5:**
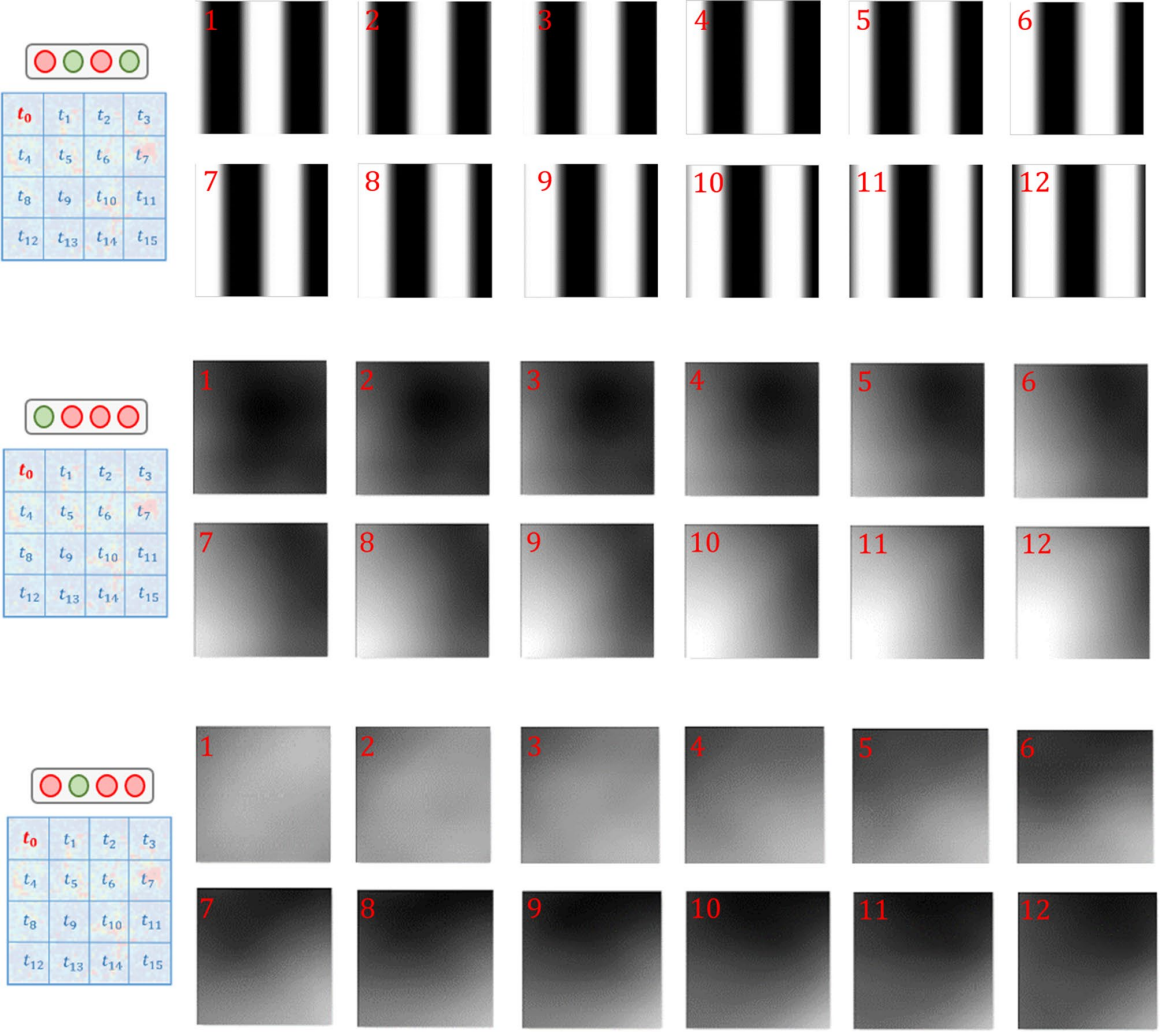
Results related to Experiment 4. Here are reported, from top to bottom, the visualizations of one hidden state and two different hidden units from a trained cRBM. As detailed in text, the visualization technique is applied in the same way as for the mcRBM, but in this case—having more visible layers, as pictured in Fig. 1—we have several averages, related to different time frames. In this case, for visualization purposes, we cut the portion of stimuli associated with the patch used to carry out our analysis *(t0)*. The red numbers indicate the temporal evolution (1 stands for the most delayed frame)

Finally, we report results obtained by modeling data recorded for *Experiment 1* analyzed with cRBMs, to understand whether also the temporal evolution of the stimuli can be decoded. The only difference in the way data are fed to the model is that a sequence of time-delayed samples from retinal activity are given in input to this particular RBM, instead of single occurrences. An additional parameter is thus the length of history modeled. We decided to design the model in such a way that the most delayed visible layer is fed with a firing rate sample associated with a stimulus which is in counterphase with respect to the present one. We arbitrarily chose to use 12 evenly spaced visible layers.

Figure 5 shows visualizations associated with two different binary units of a model trained with neurons from the patch t0. The red numbers in the squares indicate the temporal evolution (the smaller the number, the more delayed the visible layer). For visualization purposes, in this case we crop the portions of stimuli associated with the portion of retina analyzed.

## Discussion

Results from *Experiment 1* demonstrate that regularities in the population activity related to simple stimuli shown to the retina can be discovered by modeling the joint distribution with a mcRBM. Indeed, the interpretation of results reported in the first two columns of Fig. 2 is straightforward: latent states encode regularities in RGC joint distribution associated with different phases of the stimuli proposed to the retina. This is a key result in our study, since one goal was to figure out if such information could be recovered from the population activity in an unsupervised fashion using this class of models.

As can be observed, STA-based representations are slightly more blurred than original stimuli, due to the fact that similar stimuli are averaged. This is a positive aspect of the results, since we can expect the population activity related to one stimulus to be more similar to the activities related to comparable stimuli, rather than activities associated with very different stimuli. The consequence of this similarity is that firing rate samples associated with similar stimuli are described by the same binary states. We observed that increasing the size of the mcRBM hidden state leads to less blurred STA-based representations. This is an obvious consequence, since the machine can learn up to $2^N$ different states, where *N* is the number of hidden units. If *N* is big, the model can learn a number of regularities big enough to learn each single input configuration.

Our interpretation of results related to hidden units (Fig. 2, columns 3 and 4) is that they encode activities associated with population receptive fields. Indeed, also Prentice et al.^1^ showed that latent factors of a Hidden Markov Model can model activities that are the sum of different receptive fields. It is worth noting that the worst results, in the sense of more noisy images, are the ones related to patch t7 (Fig. 2, third row): indeed, this portion of the chip is the one with less neurons recorded (61). This finding might suggest that modeling the distribution resulting from many different neurons is more informative.

Results from *Experiment 3* show that even when stimulated with more complex protocols (specifically, images of a brick wall), the retinal population activity can be efficiently modeled with RBMs. Indeed, this experiment shows that single latent variables encode distinct visual features (see Fig. 4b). These results show that the joint distribution of RGC represent a reliable code also with less trivial stimuli, and the modes of the joint distribution can be retrieved using the mcRBM.

Figure 3 shows that well known physiological properties of the retina are reflected by the model (Experiment 2). Panel (a) shows that forcing an impairment in the retinal circuitry results in less informative models, namely models where the Mutual Information between the modes encoded by the binary states and the visual stimuli is reduced, in particular for stimuli with higher spatial frequency. Panels (b) and (c) show empirical proofs of this statement, reporting the distribution of the stimuli associated with a particular hidden state for both a non-impaired retina and an impaired retina, respectively. While in normal conditions the modes are associated with very precise stimuli, after the impairment the states are associated with stimuli in a pathological way. In this particular case, stimuli in counterphase are encoded in the same mode, showing that blocking GABA receptors has a dramatic effect on retinal behavior.

Finally, Fig. 5 shows results related to *Experiment 4*. The hidden states units of a cRBM can learn the modes in the distribution associated with temporal patterns that are related to well defined dynamics of the stimuli. In particular, in the example related to the hidden state (top), it can be observed that the mc-RBM encode the patterns associated with grating motion;in the two examples related to hidden units (middle and bottom), it can be observed that the evolution of the stimuli consists in a *black-to-white* or *white-to-black* transition – which generalizes different grating dynamics. In general, the main modes in the distribution that we could retrieve were the ones associated with color transitions. Figure 5 shows indeed that the model we used can perform this task efficiently. Finally, we report here that the single *states* respond to more precise dynamics of the stimuli. Summarizing, the relationship between hidden units and states of cRBMs is the same as the one we described for mcRBMs (Fig. 2).

## Conclusion

We propose a new method to model RGC neuronal signals recorded from a large population of cells, and relate the stimuli shown to the retinal to a learned latent representation.

We evaluate our approach with different protocols: the first experiment, with heterogeneous gratings, shows that stimuli and their temporal evolution can be retrieved from RGC activity. The experiment on pharmacological blockage of GABA receptors shows that physiological properties of the retina are preserved by these models. Finally, the natural scene experiment demonstrates that also more complex stimuli can be retrieved from the signals.

Further investigations to better understand the applicability of Restricted Boltzmann Machines modelling in decoding large-scale spiking activity recordings in brain circuits are warranted. In perspective, this approach might support the development of new generations of brain machine interfaces for clinical applications.

## Supplementary information

Supplementary file1

## Data Availability

The datasets generated to carry out the experiments reported in this work are available from the corresponding author on request.
