# Supplementary material for: Modeling a population of retinal ganglion cells with restricted Boltzmann machines
Source: Sci Rep. 2020 Oct 6;10:16549. doi: 10.1038/s41598-020-73691-z (PMC7538558; doi:10.1038/s41598-020-73691-z)
Supplement: Supplementary file 1 — Supplementary file1 [file 41598_2020_73691_MOESM1_ESM.pdf]

Supplementary Material for the Submission "Modeling a Population of Retinal Ganglion Cells with Restricted Boltzmann Machines"

Riccardo Volpi[1,+,*], **Matteo Zanotto**[1,+], **Alessandro Maccione**[1], **Stefano Di Marco**[2], **Luca Berdondini**[1], **Diego Sona**[1], **and Vittorio Murino**[1,3]

[1]Istituto Italiano di Tecnologia
[2]Università degli Studi dell'Aquila
[3]Università di Verona
[*]riccardo.volpi@iit.it
[+]these authors contributed equally to this work

ABSTRACT

We report here the supplementary information that accompanies the submission. In particular, it is related to the mathematical beckground for the Mean-covariance Restricted Boltzmann Machine model.

The Mean-covariance Restricted Boltzmann Machine

The mcRBM model[1,2] is a modified version of the standard RBM, where the hidden units are divided in two different sets: mean units and precision units. The former is used to model the mean value of the inputs, the latter to explicitly modeling the covariance between the observed variables. The presence of this second set allows to obtain a much better fit of the data distribution than what can be achieved with simpler models like Gaussian RBMs. The energy function is thus divided in two different terms: $E_{mc} = E_m + E_c$, where the former (energy of mean units) is defined by (3) and the latter (energy of precision units) is defined by

$$E_c(\mathbf{v}, \mathbf{h}_c) = -\mathbf{d}^T \mathbf{h}_c - (\mathbf{v}^T \mathbf{R})^2 \mathbf{P} \mathbf{h}_c, \tag{1}$$

where $\mathbf{R}$ is the visible-factor weight matrix, $\mathbf{P}$ the factor hidden pooling matrix, and $\mathbf{d}$ is the hidden bias vector. The conditional distribution of the hidden units given the visible ones is then

$$P(\mathbf{h}|\mathbf{v}) = \sigma\Big(\mathbf{d} + \big(\mathbf{v}^T \mathbf{R}^2 \mathbf{P}\big)^T\Big). \tag{2}$$

The conditional distribution of the visible units given the hidden ones is given by

$$P(\mathbf{v}|\mathbf{h}_c, \mathbf{h}_m) \propto N(\Sigma \mathbf{W} \mathbf{h}_m, \Sigma), \tag{3}$$

where

$$\Sigma = \big(\mathbf{R}(diag\big(-\mathbf{P}^T \mathbf{h}_c)\big)\mathbf{R}^T\big)^{-1}. \tag{4}$$

An important property of mcRBMs is that, conditioning on the latent variables, the observed ones are approximately jointly Gaussian distributed[2]. The mean and covariance of these Gaussians are defined by the specific values of the hidden units. This means that each of the possible binary vectors representing the latent variable values is associated with one mode of the joint distribution of the inputs. This aspect makes mcRBM a very good model for our purposes of finding the regularities associated with visual stimuli shown to the retina.

Acknowledgments

This research received financial support from the 7th Framework Programme for Research of the European Commission, under Grant agreement no. 600847: RENVISION project of the Future and Emerging Technologies (FET) programme Neuro-bio-inspired systems (NBIS) FET-Proactive Initiative.

Author contributions statement

RV wrote the manuscript. All authors are aware of the content of the manuscript and helped improving it through multiple revisions. MZ designed and carried out the experiments related to the mc-RBM model. RV designed and carried out the experiments related to the cRBM model. AM, SDM and LB performed the MEA data acquisition. VM, DS and LB conceived the project. VM coordinated the project.

Competing interests statement

The authors declare that no competing interests exist.

Data availability statement

The datasets generated to carry out the experiments reported in this work are available from the corresponding author on request.

References

1. George Dahl, Marc'Aurelio Ranzato, Abdel-Rahman Mohamed and Geoffrey E. Hinton Phone recognition with the mean-covariance restricted Boltzmann machine. Advances in Neural Information Processing Systems (NIPS), 2010.
2. Marc-Aurelio Ranzato and Geoffrey E. Hinton Modeling Pixel Means and Covariances Using Factorized Third-Order Boltzmann Machines. In Computer Vision and Pattern Recognition, 2010
